# Effects of 7,8-Dihydroxyflavone on Lipid Isoprenoid and Rho Protein Levels in Brains of Aged C57BL/6 Mice

**DOI:** 10.1007/s12017-020-08640-0

**Published:** 2020-12-30

**Authors:** Sarah Ötzkan, Walter E. Muller, W. Gibson Wood, Gunter P. Eckert

**Affiliations:** 1 Department of Pharmacology, Biocenter Niederursel, University of Frankfurt, Goethe-University, Max-von-Laue-St. 9, 60438 Frankfurt, Germany; 2 Department of Pharmacology, Geriatric Research, Education and Clinical Center, University of Minnesota School of Medicine, VAMC, Minneapolis, MN 55417 USA; 3 Institute of Nutritional Sciences, Laboratory for Nutrition in Prevention and Therapy, Justus-Liebig-University of Giessen, Biomedical Research Center Seltersberg (BFS), Schubertstr. 81, 35392 Giessen, Germany

**Keywords:** Isoprenoids, Cholesterol, Brain aging, Rho proteins, Rac1, Rab3A, 7,8-dihydroxyflavone, Tiam1, BDNF

## Abstract

Synaptic impairment may be the main cause of cognitive dysfunction in brain aging that is probably due to a reduction in synaptic contact between the axonal buttons and dendritic spines. Rho proteins including the small GTPase Rac1 have become key regulators of neuronal morphogenesis that supports synaptic plasticity. Small Rho- and Ras-GTPases are post-translationally modified by the isoprenoids geranylgeranyl pyrophosphate (GGPP) and farnesyl pyrophosphate (FPP), respectively. For all GTPases, anchoring in the plasma membrane is essential for their activation by guanine nucleotide exchange factors (GEFs). Rac1-specific GEFs include the protein T lymphoma invasion and metastasis 1 (Tiam1). Tiam1 interacts with the TrkB receptor to mediate the brain-derived neurotrophic factor (BDNF)-induced activation of Rac1, resulting in cytoskeletal rearrangement and changes in cellular morphology. The flavonoid 7,8-dihydroxyflavone (7,8-DHF) acts as a highly affine-selective TrkB receptor agonist and causes the dimerization and autophosphorylation of the TrkB receptor and thus the activation of downstream signaling pathways. In the current study, we investigated the effects of 7,8-DHF on cerebral lipid isoprenoid and Rho protein levels in male C57BL/6 mice aged 3 and 23 months. Aged mice were daily treated with 100 mg/kg b.w. 7,8-DHF by oral gavage for 21 days. FPP, GGPP, and cholesterol levels were determined in brain tissue. In the same tissue, the protein content of Tiam1 and TrkB in was measured. The cellular localization of the small Rho-GTPase Rac1 and small Rab-GTPase Rab3A was studied in total brain homogenates and membrane preparations. We report the novel finding that 7,8-DHF restored levels of the Rho proteins Rac1 and Rab3A in membrane preparations isolated from brains of treated aged mice. The selective TrkB agonist 7,8-DHF did not affect BDNF and TrkB levels, but restored Tiam1 levels that were found to be reduced in brains of aged mice. FPP, GGPP, and cholesterol levels were significantly elevated in brains of aged mice but not changed by 7,8-DHF treatment. Hence, 7,8-DHF may be useful as pharmacological tool to treat age-related cognitive dysfunction although the underlying mechanisms need to be elucidated in detail.

## Introduction

Synaptic impairment may be the main cause of cognitive dysfunction in brain aging instead of neuronal loss (Burke and Barnes 2006; Grillo et al. 2013; Morrison and Baxter 2012). Age-related synaptic dysfunction is probably due to a reduction in synaptic contact between the axonal buttons and dendritic spines (Hof and Morrison 2004; Mostany et al. 2013). Rho proteins including the small GTPase Rac1 have become key regulators of neuronal morphogenesis that supports synaptic plasticity (Gonzalez-Billault et al. 2012). A vast majority of small Rho-GTPases including Rac1 are post-translationally prenylated by the isoprenoid geranylgeranyl pyrophosphate (GGPP) using geranylgeranyl transferase-I (GGTase-I). We published that a decrease in membrane-associated Rho proteins in the brains of aged mice is associated with a downregulation of GGTase-Iβ and an upregulation of GGPP. We suggested that the downregulation of GGTase-Iβ may be one of the mechanisms that cause age-related weakening of synaptic plasticity (Afshordel et al. 2014). Directly related to our findings Hottman et al. recently published that systemic or forebrain neuron-specific deficiency of GGTase-I reduces dendritic spine density and impairs synaptic plasticity in the brains of young adult mice, concurrently with reduced geranylgeranylation of Rho proteins (Hottman et al. 2018).

For all GTPases, anchoring in the plasma membrane is essential for their activation by guanine nucleotide exchange factors (GEFs). GEFs catalyze the exchange of GDP to GTP in a complex multi-step reaction and thus cause the activation of the corresponding GTPase (Schmidt and Hall 2002). GEFs receive an upstream signal that triggers a specific signal transduction cascade in which the Rho-GTPases are involved. Before activation of the GTPase, the specific GEF is recruited to the plasma membrane or a receptor. There, in the initial step, a complex of GDP-bound GTPase and GEF is formed, which has only a low-binding affinity. This complex is converted into a stable nucleotide-free complex of GTPase and GEF by the dissociation of GDP. Subsequent binding of GTP to the GTPase dissolves the complex, resulting in the active GTP-bound form of the GTPase (Schmidt and Hall 2002).

Rac1-specific GEFs include the protein T lymphoma invasion and metastasis 1 (Tiam1) (Kiraly et al. 2010), which is associated with the development of synapses and their plasticity and is necessary for the development of dendritic spines (Tolias et al. 2011). Tiam1 is associated with numerous neuronal processes such as neuronal migration, neurite growth, and axonal specification (Kawauchi et al. 2003; Kunda et al. 2001; Leeuwen et al. 1997) and interacts with the TrkB receptor to mediate the brain-derived neurotrophic factor (BDNF)-induced activation of Rac1, resulting in cytoskeletal rearrangement and changes in cellular morphology (Miyamoto et al. 2006; Zhou et al. 2007).

BDNF is derived from its precursor, pro-BDNF representing a dimeric protein with a molecular weight of about 28 kDa, which is composed of two non-covalently bound subunits. Each subunit has a molecular weight of about 14 kDa and is structurally related to NGF (Rosenthal et al. 1991). In the CNS, BDNF is found to a high degree in the hippocampus, but also in the amygdala, thalamus, the approaches of the olfactory system, the inner and outer pyramidal levels of the neocortex, the claustrum, the septum, the cerebellum, and the superior colliculi in pyramidal and granule cells (Connor and Dragunow 1998). BDNF is localized in neuronal and glial cells—in the latter to a smaller extent (De Pins et al. 2019; Saha et al. 2006).

**Fig. 1:**
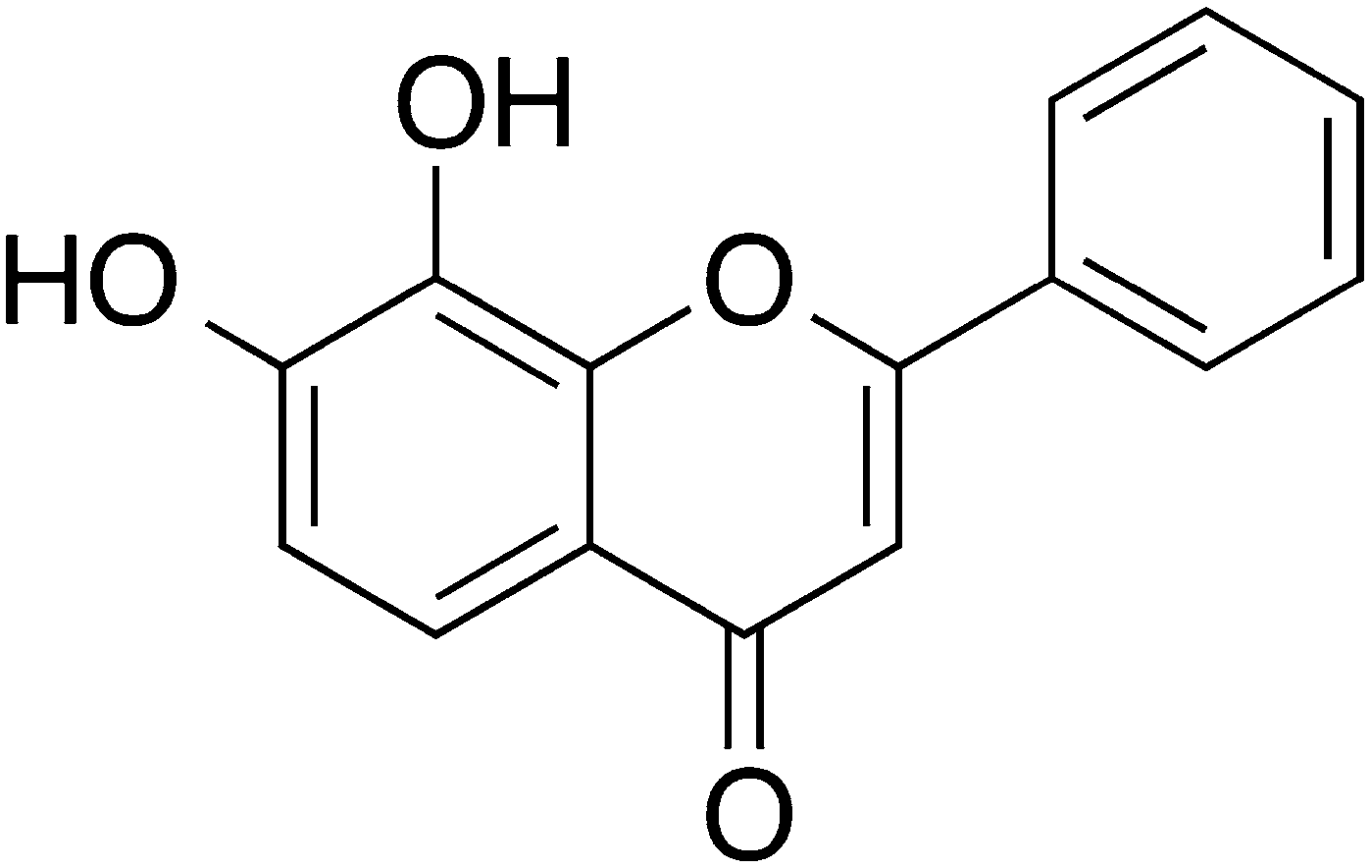
Structure of 7,8-Dihydroxyflavone (7,8-DHF)

The flavonoid 7,8-dihydroxyflavone (7,8-DHF, Fig. 1) is part of the family of polyphenolic compounds and occurs naturally in *Godmania aesculifolia*, *Tridax procumbens,* and in leaves of Primroses (Du and Hill 2015). 7,8-DHF acts as a highly affine-selective TrkB receptor agonist and causes the dimerization and autophosphorylation of the TrkB receptor and thus the activation of downstream signaling pathways (Jang et al. 2010). Thus, 7,8-DHF has the same neurotrophic properties as BDNF and was proposed as useful for treating various BDNF-implicated human disorders including Alzheimer’s disease (Chen et al. 2018; Liu et al. 2016). The flavone 7,8-DHF is metabolized and the conjugated forms are the main metabolites in monkey plasma (Sun et al. 2017). 7,8-DHF crosses the blood–brain barrier, binds selectively to TrkB receptors, and activates them, even in the absence of endogenous BDNF (Jang et al. 2010). Thus, 7,8-DHF mediates neuronal survival, differentiation, synaptic plasticity, and neurogenesis (Andero et al. 2011; Chen et al. 2014; Tsai et al. 2013; Zeng et al. 2012; Zhang et al. 2014). 7,8-DHF has also been successfully been applied in models of alcohol-related behavior, rotenone-induced neurotoxicity in rodents, and high glucose-damaged neuronal cells (Cho et al. 2019; Li et al. 2020; Nie et al. 2019).

In the current study, we investigated the effects of 7,8-DHF on cerebral lipid isoprenoid and Rho protein levels. In analogy to the preceding study on the influence of brain aging on Rho- and Rab-GTPase levels, male C57BL/6 mice aged 3 and 23 months were used (Afshordel et al. 2014). Aged mice were daily treated with 100 mg/kg b.w. 7,8-DHF by oral gavage for 21 days. FPP, GGPP, and cholesterol levels were determined in brain tissue. In the same tissue, the content of Tiam1 and TrkB in was measured. The cellular localization of the Rho-GTPase Rac1 and Rab-GTPase Rab3A was studied to draw conclusions on the activity and functionality of these Rho- and Rab-GTPases. For this purpose, the total homogenate of the brain tissue of the test animals as well as preparations of membrane fractions was analyzed.

## Materials and Methods

### Chemicals and Reagents

7,8-Dihydroxyflavone hydrate (#D5446; purity > 98%; CAS Number 38183-03-8)) was purchased from Sigma-Aldrich (Darmstadt, Germany); ammonium hydroxide solution 28–30% was purchased from Alfa Aesar (Karlsruhe, Germany); and the phosphatase inhibitors Halt® and Phosstop® from Thermo-Fisher/Piercenet (Bonn, Germany) and Roche Diagnostics GmbH (Mannheim, Germany). All solvents were of analytical grade or higher quality. Acetonitrile was obtained from Carl Roth GmbH (Karlsruhe, Germany), 1-butanol, n-hexane, 2-propanol, methanol, acetone, ammonium acetate and assay buffer compounds: Tris–HCl, MgCl_2_, ZnCl_2_ and Na_2_CO_3_ were obtained from Merck (Darmstadt, Germany). Octyl-β-d-glucopyranoside and dithiothreitol were from Sigma-Aldrich (Schnelldorf, Germany). Millipore water was used for all solutions (Schwalbach, Germany).

### Animals

Male C57BL/6 mice (3 and 23 months of age) were obtained from Janvier (St. Berthevin Cedex, France). The mice were maintained on a 12-h dark–light cycle with pelleted food and tap water ad libitum. In the design of the experiments, the ARRIVE guidelines were followed. All experiments were carried out by individuals with appropriate training and appropriate experience in accordance with the European Communities Council Directive (86/609/EEC) and the ARRIVE guidelines. Experiments were approved by the regional authority (Regierungspraesidium Darmstadt, #FU1062). The animals treated once daily by oral gavage at the same time of day with 100 mg 7,8-DHF/kg body weight for a total of 21 days. The animals were weighed before each administration and treated with the corresponding volume of 7,8-DHF solution. The control animals were treated with the vehicle solution in the same way. Throughout the feeding study, the weights of the test animals were determined and recorded once daily to monitor the physical condition of the animals. 7,8-DHF was dissolved in 0.2% agarose solubilized in H_2_O (75 mg/3 ml). The vehicle (0.2% agarose in H_2_O) served as control.

### Brain Tissue Preparation

Brains were dissected into two hemispheres (without brain stem and cerebellum), snap frozen in liquid nitrogen and stored at − 80 °C until use. For lipid analysis, one entire hemisphere was used. For the Western blot analysis, the second hemisphere was used.

### Protein Analysis

Protein levels were measured using the BCA Protein Assay Kit from Thermo-Scientific/Pierce (Bonn, Germany). Samples were measured in triplicates.

### Membrane Isolation

Brain membrane and cytosolic fractions were isolated as reported (Afshordel et al. 2014). Briefly, tissue samples were sequentially processed by homogenization and ultracentrifugation (100,000 g for 20 min) to obtain supernatants (TBS, soluble-cytosol fraction). Pellets were then sonicated in lysis buffer and again centrifuged to obtain lysis extract supernatants (membrane-cytoskeletal extract).

### Western Blot Analysis

Western blot analysis was performed as described (Afshordel et al. 2014). For specific protein determination, samples were prepared by diluting (in total cell or brain tissue homogenate: 10 µg for BDNF, TrkB, Tiam1, Rac1, and Rab3A protein; in membrane preparations: 80 µg for Rac1 and Rab3A protein) with the reducing agent (10x) and NuPAGE LDS sample buffer (4×). After denaturation for 10 min at 95 °C, the samples were electrophoretically separated on a 4–12% NuPAGE Bis/Tris gel (Invitrogen, Germany) for 40 min at 190 V and then transferred on a PVDF membrane for 90 min at 30 V and blocked with 7.5% non-fat-dried milk in Millipore water for 30 min. Membranes were incubated with primary antibodies (anti-TrkB, #sc-12; anti-Tiam1, #sc-872; anti-Rac1, #sc-95; anti-Rab3A, #sc-308; anti-BDNF, #sc-546 Santa Cruz, Heidelberg, Germany; anti-Flotillin-1, BD Biosciences, Heidelberg, Germany, #610820; anti-GAPDH, #MAB374, Millipore, Schwalbach). Band analysis was performed using BioRad’s Quantity One Software.

### Isoprenoid and Cholesterol Analysis

Determination of GGPP and FPP levels in mouse brain homogenates was performed using a validated HPLC-FD method as previously described (Hooff et al. 2010a). Cholesterol levels were determined spectrophotometrically using an colorimetric enzymatic test as described previously (Franke et al. 2007).

### Statistics

All data are expressed as means ± standard error of the mean (SEM) unless stated otherwise. For direct comparison of differences between two and three groups, student’s *t*-test and one-way ANOVA followed by Tukey’s post-test were calculated, respectively. All calculations were performed with GraphPad Prism version 5.00 for Mac, GraphPad software, San Diego, USA.

## Results

Similar to the previous study on the impact of aging on brain Rho- and Rab-GTPase levels (Afshordel 2010), male C57BL/6 mice aged 3 and 23 months were used as an animal model to study the effects of 7,8-DHF (Fig. 1) on cerebral lipid isoprenoid and Rho protein levels in aged mice.

### 7,8-DHF Restores Rac1- and Rab3A- Levels in Synaptic Plasma Membranes Isolated from Brains of Aged Mice

The cellular localization of the Rho-GTPase Rac1 and Rab-GTPase Rab3A was investigated to allow conclusions on the activity and functionality of these Rho- and Rab-GTPases. For this purpose, the total homogenate of the brain tissue of the test animals as well as preparations of synaptosomal membrane fractions was analyzed.

**Fig. 2:**
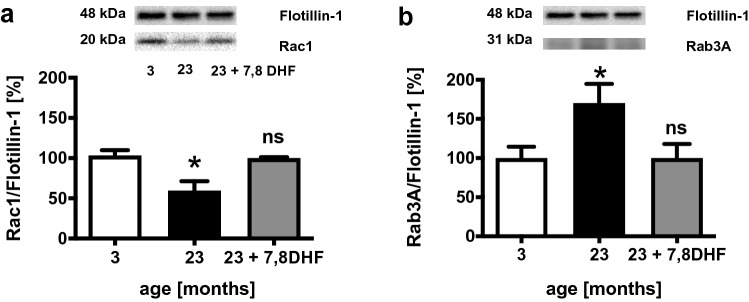
Brain membrane-associated Rac1 and Rab3A protein levels. Western blot analysis of brain membrane preparations isolated from brains of young (3-month old, 3), aged (23-month old, 23), and aged mice treated with 7,8-dihydroxyflavone (23 + 7,8-DHF) was used to characterize the prenylation of **a** Rac1 and **b** Rab3A. Levels of membrane-associated, geranylgeranylated Rho- and Rab-GTPases were normalized to the membrane marker Flotillin1. Each graph shows representative western blots. Mean ± SEM, ANOVA (p* < 0.05); ns, not significant compared to controls (3-month old), *n* = 6

Membrane-bound prenylated Rac1 showed a significant reduction in brain tissue of 23-month-old C57BL/6 mice compared to the 3-month old animals (Fig. 2a). There was no difference between the content of Rac1 in the total brain tissue homogenate of the 23-month-old mice and the 3-month-old C57BL/6 mice (data not shown). Feeding of 7,8-DHF to the 23-month-old C57BL/6 mice induced a significant shift (p*** < 0.001) in the cellular localization of Rac1 toward an increase in the protein content of prenylated membrane-bound Rac1 to the level of the brain preparations of the 3-month-old animals (Fig. 2a). The protein content of Rac1 in the total homogenate of the brains of 23-month-old C57BL/6 mice fed with 7,8-dihydroxyflavone showed no difference to the 3- and 23-month-old control animals (data not shown).

The content of the Rab3A protein expressed in the central nervous system was also measured in the brains of 3- and 23-month-old C57BL/6 mice. No difference was detected in the total homogenate of brain tissue between the two age groups (data not shown). Analysis of the levels of membrane-bound Rab3A in the brains of 23-month-old mice showed a significant increase compared to the 3-month-old mouse brains (Fig. 2b). Feeding the 23-month-old C57BL/6 mice with 7,8-DHF resulted in a non-significant reduction (*p* = 0.06) of membrane-bound Rab3A levels in the brain preparations compared to the untreated 23-month-old animals (Fig. 2b).

### 7,8-DHF Restores Tiam1- Levels in Brain Tissue Isolated from Aged Mice

In order to elucidate the cellular mechanism behind the action of the 7,8-dihydroxyflavone, Western blot analyses of the proteins TrkB, BDNF, and Tiam 1 were performed with the total brain homogenate of the experimental animals.

**Fig. 3:**
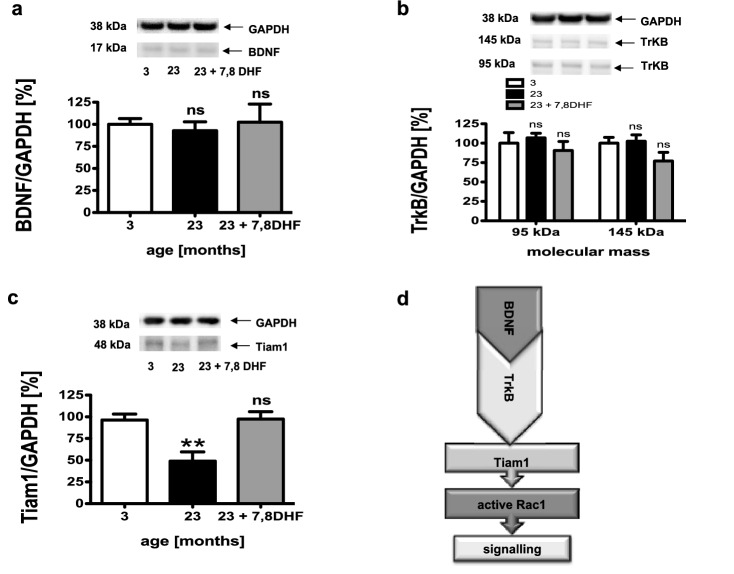
Brain BDNF, TrkB, and Tiam1 protein levels. Western blot analysis of brain total homogenates isolated from brains of young (3-month old, 3), aged (23-month old, 23), and aged mice treated with 7,8-dihydroxyflavone (23 + 7,8-DHF) was used to measure the levels of (**a**) brain-derived neurotrophic factor (BDNF), of (**b**) Tyrosine kinase B receptor (TrkB), and of (**c**) the T lymphoma invasion and metastasis guanosine exchange factor 1 (Tiam1). Protein levels were normalized to GAPDH. Each graph shows representative Western blots. Mean ± SEM, ANOVA (p** < 0.01); ns, not significant, compared to controls (3-month old), *n* = 6. (**d**) The TrkB receptor binds its ligand BDNF and the interaction with the Rac1-specific GEF Tiam1 mediates the BDNF-induced activation of Rac1. This leads to a rearrangement of the cytoskeleton and changes in the cellular morphology of the affected neurons (Miyamoto et al. 2006; Zhou et al. 2008a, b)

By activating the TrkB receptor, BDNF regulates axonal orientation, synaptic functions, synaptogenesis, and neuronal differentiation. In this context, BDNF is able to activate various Rho-GTPases including Rac1 by activating the TrkB receptor (Fig. 3d). The investigation of the protein content of BDNF showed no difference between the brains of the three experimental animal groups (Fig. 3a). The full-length receptor TrkB (145 kDa) mediates signals that mediate neuronal survival and differentiation and synaptic plasticity. These effects are inhibited by the truncated isoforms truncated TrkB and TrkB-Shc (95 kDa) by forming heterodimers with the full-length TrkB (Wong et al. 2013). Neither the protein content of the full-length TrkB (145 kDa) nor the protein content of the truncated isoforms of TrkB (95 kDa) showed a difference between the brains of 3-month-old C57BL/6 mice, 23-month-old animals, and 23-month-old C57BL/6 mice fed with 7,8-DHF. Tiam1 is a Rac1-specific GEF that interacts with the TrkB receptor to mediate the BDNF-induced activation of Rac1 resulting in cytoskeletal rearrangement and changes in cellular morphology (Fig. 3d) (Miyamoto et al. 2006; Zhou et al. 2007). The protein content of Tiam1 showed a significant reduction in the brains of 23-month-old C57BL/6 mice compared to 3-month-old animals (Fig. 3c). Feeding of 7,8-DHF to the 23-month-old C57BL/6 mice resulted in an non-significant increase (*p* = 0.052) in the protein content of Tiam1 to the level of the 3-month-old animals (Fig. 3c).

### 7,8-DHF has no Effects on Brain Isoprenoids and Cholesterol Levels

**Fig. 4:**
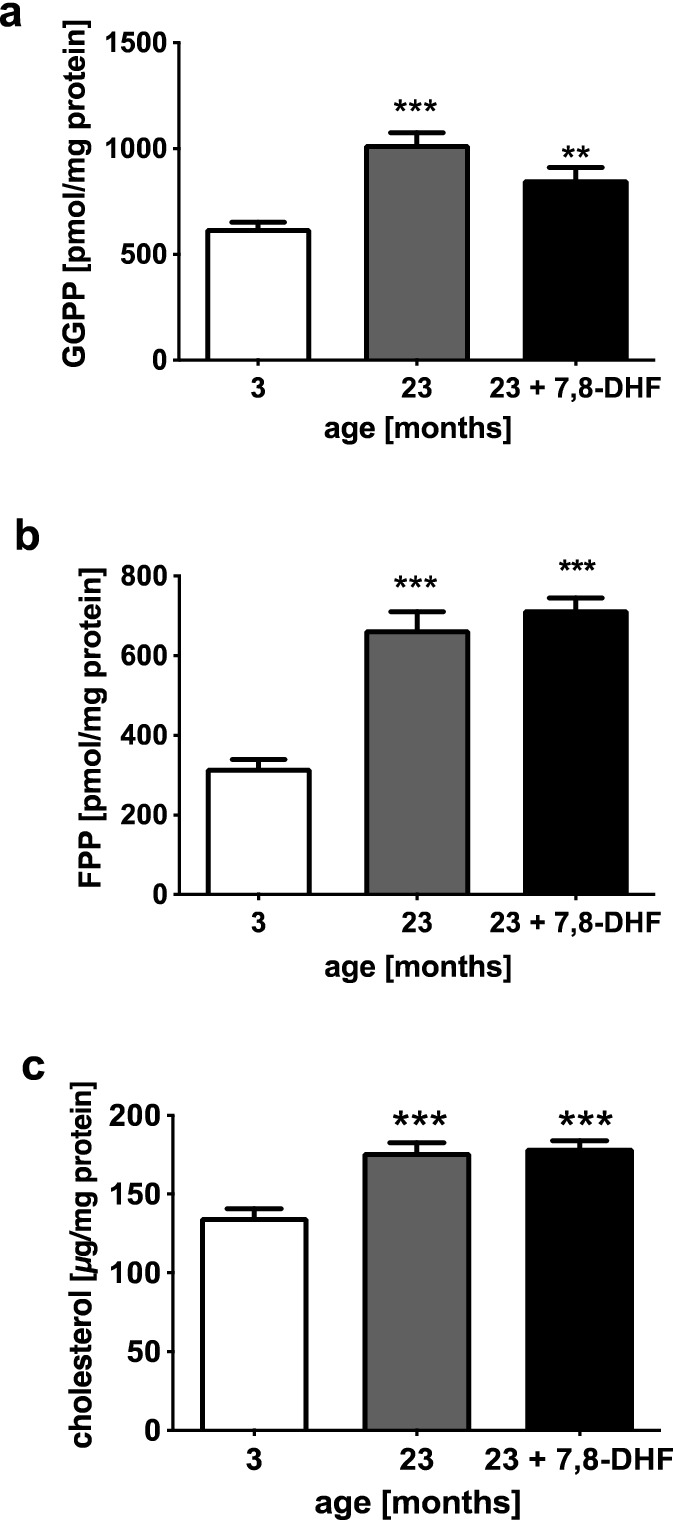
Brain GGP-, FPP-, and cholesterol levels. Levels of geranylgeranyl- (**a**, GGPP) and farnesyl pyrophosphate (**b**, FPP) were determined using a HPLC method, cholesterol levels (**c**) were determined using an enzymatic method in brain tissue isolated from young (3-month old, 3), aged (23-month old, 23), and aged mice treated with 7,8-dihydroxyflavone (23 + 7,8-DHF). Mean ± SEM, ANOVA (p** < 0.01; p*** < 0.001), compared to controls (3-month old), *n* = 8

Investigations of brain tissue of C57BL/6 mice revealed age-related changes in all three brain lipids investigated. GGPP, FPP, and cholesterol levels were significantly higher in the brains of aged mice compared to the young animals (Fig. 4a–c). Feeding the C57BL/6 mice with 7,8-dihydroxyflavone only resulted in a non-significant reduction (*p* = 0.06) of the isoprenoid GGPP in the brains of the aged animals (Fig. 4a).

## Discussion

Aging is associated with cognitive deficits related to synaptic plasticity (Burke and Barnes 2010; Petralia et al. 2014; Shetty and Sajikumar 2017). The changes in dendritic branching and spine density underlying the age-related decrease in synaptic plasticity have been widely studied. However, the mechanisms for these changes are not yet well understood (Burke and Barnes 2006; Petralia et al. 2014). The actin cytoskeleton plays a crucial and essential role in controlling the development and maintenance of spines and synapses (Hu et al. 2016; Tolias et al. 2011), whose organization and function depend on proteins of the Rho family (Bongmba et al. 2011; Chen et al. 2012; Newey et al. 2005; Tolias et al. 2011). Normal function of these proteins requires attachment of 20-carbon GGPP to the cysteine residue of a carboxy terminal CAAX motif. Prenylated proteins may undergo further posttranslational modifications, all of which increase the hydrophobicity of the proteins and facilitate membrane association required for their active state (reviewed by (Boulter et al. 2012; Hooff et al. 2010c; McTaggart 2005; Samuel and Hynds 2010)).

We found a switch in the localization of Rac1 to lowered concentrations in membranes in the brain of aged mice compared to younger mice, which is consistent with our previous studies on Rho-GTPases in the brains of aged mice (Afshordel et al. 2014). We have previously shown that the age-related decrease in membrane-associated Rho proteins is associated with a decrease in the activity of GGTase-I, which regulates the binding of GGPP to Rho-GTPases. In vitro GGTase-I inhibition mimicked the changes we observed in the brain of aged mice, including a decreased frequency of synaptic markers (Afshordel et al. 2014). That GGTase-I deficiency leads a significant decrease in cortical spine density and cognitive function has been recently shown in GGT-haplodeficient mice (Hottman et al. 2018). Using a conditional forebrain neuron-specific GGT knockout they further demonstrated a decrease of both the magnitude of hippocampal LTP and the dendritic spine density of cortical neurons in those mice (Hottman et al. 2018). We have shown that the decrease in membrane-associated Rho proteins was unique to those proteins that are prenylated by the transferase GGTase-I, but not to Rab proteins prenylated by GGTase-II (Afshordel et al. 2014). This was confirmed in the current study. Accordingly, a significant reduction of cell membrane-associated (geranylgeranylated) Rac1 and RhoA but not (farnesylated) H-Ras was demonstrated in mice harboring a conditional forebrain neuron-specific GGT-knockout (Hottman et al. 2018). We observed elevated (geranylgeranylated) Rab3A protein levels in membrane preparations isolated from aged brain (Afshordel et al. 2014). Rab3A is prenylated by Rab-GTPase and the protein is associated with the membrane of synaptic vesicles and is involved in controlling the targeting or docking of these vesicles to the presynaptic membrane for the release of neurotransmitters (Stettler et al. 1994). The expression of the Rab3A gene decreases significantly with age (Saetre et al. 2011). We cannot explain this apparent contrast between protein levels and gene expression. However, too high Rab3A protein levels might lead to the downregulation of the gene expression. This assumption would have to be verified experimentally in following studies. Whether our current findings contribute to age-related synaptic dysfunction needs further investigation. However, it is important to note that 7,8-DHF also restored Rab3A membrane levels in treated aged mice.

Li et al. showed that GGTase-I mediates synaptogenesis by BDNF-induced Rac1 activation (Li et al. 2013), which is directly related to our data. BDNF belongs to the neurotrophins, small secreted proteins that promote the growth, differentiation, and survival of neurons in the central and peripheral nervous system regulate neuronal morphology and also synaptic transmission and plasticity (Schuman 1999; Skaper 2005). BDNF binds to various neurotrophin receptors, including specifically to the TrkB receptor and is able to activate it (Berkemeier et al. 1991). Although different neurotrophins bind to the TrkB receptor, it is able to discriminate the neurotrophins from each other and to activate different signaling cascades depending on their binding (Lewin and Barde 1996). BDNF is a necessary factor for the branching of axons and dendrites and thus promotes the formation and maturation of synapses (Alsina et al. 2001). BDNF also acts as a modulator of synaptic function and plasticity (Park and Poo 2013). The interaction of the TrkB receptor with the Rac1-specific GEF Tiam1 mediates the BDNF-induced activation of Rac1 (Fig. 3d). This leads to a rearrangement of the cytoskeleton and changes in the cellular morphology of the affected neurons (Miyamoto et al. 2006; Zhou et al. 2007). We observed that cerebral levels of BDNF and of TrkB were equal in brain tissue isolated from young and aged mice. Moreover, BDNF and TrkB levels were not changed in brains of aged mice after 7,8-DHF treatment. However, we report the novel finding that brain levels of Tiam1 were significantly reduced in brains of aged mice. Importantly, treatment of aged mice with 7,8-DHF restored Tiam1 and also the levels of membrane-bound Rac1 and Rab3A in brains of aged mice.

We have demonstrated that GGTase-I inhibition significantly elevated GGPP levels in SH-SY5Y cells (Afshordel et al. 2014). These in vitro results mirror what we observed in the brain tissue of aged mice and may explain the increase in GGPP levels in the brain of aged mice in this and our previous studies (Afshordel et al. 2014; Hooff et al. 2012). The age-related decrease in GGTase-I activity caused an abnormal accumulation of GGPP (see above, (Afshordel et al. 2014)). The common understanding is that GGPP is mainly responsible for protein prenylation. However, it should be noted that GGPP may have effects that are independent of protein prenylation (Miquel et al. 1998; Zhou et al. 2008a, b). Treatment of aged mice with 7,8-DHF resulted in a slight and non-significant reduction of GGPP levels. Correspondingly, levels of membrane-bound, geranylated Rac1 were significantly elevated in aged mice after treatment with 7,8-DHF. Based on previous data (Afshordel et al. 2014), it can be speculated that increased prenylation of Rac1 may have reduced the pool of GGPP in the brain tissue of aged mice. Accordingly, it has been shown that manipulation of isoprenoid and protein prenylation regulates synaptic plasticity and cognitive function in animal models (Cheng et al. 2013; Costa et al. 2002; Li et al. 2006; Mans et al. 2010, 2012; Ye and Carew 2010). However, FPP and cholesterol levels were significantly elevated in brains of aged mice as previously reported (Afshordel et al. 2014; Hooff et al. 2010b), but not changed by 7,8-DHF treatment.

In conclusion, we report the novel finding that 7,8-DHF restored levels of the Rho proteins Rac1 and Rab3A in membrane preparations isolated from brains of treated aged mice. The selective TrkB agonist 7,8-DHF did not affect BDNF and TrkB levels but restored Tiam1 levels that were found to be reduced in brains of aged mice. Hence, 7,8-DHF may be useful as pharmacological tool to treat age-related cognitive dysfunction although the underlying mechanisms need to be elucidated in detail.

## Data Availability

The dataset generated during this study is available from the corresponding author upon reasonable request.

## References

[CR1] Afshordel S, Wood WG, Igbavboa U, Muller WE, Eckert GP (2014). Impaired geranylgeranyltransferase-I regulation reduces membrane-associated Rho protein levels in aged mouse brain. Journal of Neurochemistry.

[CR2] Alsina B, Vu T, Cohen-Cory S (2001). Visualizing synapse formation in arborizing optic axons in vivo: dynamics and modulation by BDNF. Nature neuroscience.

[CR3] Andero R, Heldt SA, Ye K, Liu X, Armario A, Ressler KJ (2011). Effect of 7,8-dihydroxyflavone, a small-molecule TrkB agonist, on emotional learning. The American Journal of Psychiatry.

[CR4] Berkemeier LR, Winslow JW, Kaplan DR, Nikolics K, Goeddel DV, Rosenthal A (1991). Neurotrophin-5: A novel neurotrophic factor that activates trk and trkB. Neuron.

[CR5] Bongmba OYN, Martinez LA, Elhardt ME, Butler K, Tejada-Simon MV (2011). Modulation of dendritic spines and synaptic function by Rac1: A possible link to Fragile X syndrome pathology. Brain Research.

[CR6] Boulter E, Estrach S, Garcia-Mata R, Feral CC, Féral CC (2012). Off the beaten paths: Alternative and crosstalk regulation of Rho GTPases. The FASEB Journal.

[CR7] Burke, S. N., & Barnes, C. A. (2006). Neural plasticity in the ageing brain. *Nature Reviews Neuroscience*, *7*(1), 30–40. isi:000234139600014. 10.1038/nrn1809 16371948

[CR8] Burke SN, Barnes CA (2010). Senescent synapses and hippocampal circuit dynamics. Trends in Neurosciences.

[CR9] Chen C, Li X-H, Zhang S, Tu Y, Wang Y-M, Sun H-T (2014). 7,8-dihydroxyflavone ameliorates scopolamine-induced Alzheimer-like pathologic dysfunction. Rejuvenation Research.

[CR10] Chen C, Wang Z, Zhang Z, Liu X, Kang SS, Zhang Y, Ye K (2018). The prodrug of 7,8-dihydroxyflavone development and therapeutic efficacy for treating Alzheimer’s disease. Proceedings of the National Academy of Sciences of the United States of America.

[CR11] Chen C, Wirth A, Ponimaskin E (2012). Cdc42: An important regulator of neuronal morphology. International Journal of Biochemistry & Cell Biology.

[CR12] Cheng S, Cao D, Hottman DA, Yuan L, Bergo MO, Li L (2013). Farnesyl transferase haplodeficiency reduces neuropathology and rescues cognitive function in a mouse model of Alzheimer’s disease. The Journal of Biological Chemistry.

[CR13] Cho SJ, Kang KA, Piao MJ, Ryu YS, Fernando PDSM, Zhen AX (2019). 7,8-dihydroxyflavone protects high glucose-damaged neuronal cells against oxidative stress. Biomolecules & Therapeutics.

[CR14] Connor B, Dragunow M (1998). The role of neuronal growth factors in neurodegenerative disorders of the human brain. Brain Research Reviews.

[CR15] Costa RM, Federov NB, Kogan JH, Murphy GG, Stern J, Ohno M (2002). Mechanism for the learning deficits in a mouse model of neurofibromatosis type 1. Nature.

[CR16] De Pins B, Cifuentes-Díaz C, Thamila Farah A, López-Molina L, Montalban E, Sancho-Balsells A (2019). Conditional BDNF delivery from astrocytes rescues memory deficits, spine density, and synaptic properties in the 5xFAD mouse model of alzheimer disease. Journal of Neuroscience.

[CR17] Du X, Hill RA (2015). 7,8-Dihydroxyflavone as a pro-neurotrophic treatment for neurodevelopmental disorders. Neurochemistry International.

[CR18] Franke C, Nöldner M, Abdel-Kader R, Johnson-Anuna LN, Gibson Wood W, Müller WE, Eckert GP (2007). Bcl-2 upregulation and neuroprotection in guinea pig brain following chronic simvastatin treatment. Neurobiology of Disease.

[CR19] Gonzalez-Billault C, Munoz-Llancao P, Henriquez DR, Wojnacki J, Conde C, Caceres A (2012). The role of small GTPases in neuronal morphogenesis and polarity. Cytoskeleton (Hoboken, NJ).

[CR20] Grillo FW, Song S, Teles-Grilo Ruivo LM, Huang L, Gao G, Knott GW (2013). Increased axonal bouton dynamics in the aging mouse cortex. Proceedings of the National Academy of Sciences of the United States of America.

[CR21] Hof PR, Morrison JH (2004). The aging brain: Morphomolecular senescence of cortical circuits. Trends in Neurosciences.

[CR22] Hooff GP, Patel N, Wood WG, Müller WE, Eckert GP, Volmer DA (2010). A rapid and sensitive assay for determining human brain levels of farnesyl-(FPP) and geranylgeranylpyrophosphate (GGPP) and transferase activities using UHPLC-MS/MS. Analytical and Bioanalytical Chemistry.

[CR23] Hooff, G. P., Peters, I., Wood, W. G., Müller, W. E., Eckert, G. P., Muller, W., & Eckert, G. P. (2010b). Modulation of cholesterol, farnesylpyrophosphate, and geranylgeranylpyrophosphate in neuroblastoma SH-SY5Y-APP695 cells: Impact on amyloid beta-protein production. *Molecular Neurobiology*, *41*(2–3), 341–350. Retrieved from http://www.ncbi.nlm.nih.gov/pubmed/20405344. 10.1007/s12035-010-8117-5 20405344

[CR24] Hooff, G. P., Wood, W. G., Kim, J.-H., Igbavboa, U., Ong, W.-Y., Muller, W., et al. (2012). Brain isoprenoids farnesyl pyrophosphate and geranylgeranyl pyrophosphate are increased in aged mice. *Molecular Neurobiology*, *46*(1), 179–185. Retrieved from http://www.ncbi.nlm.nih.gov/entrez/query.fcgi?db=pubmed&cmd=Retrieve&dopt=AbstractPlus&list_uids=22692983. 10.1007/s12035-012-8285-6 22692983

[CR25] Hooff GP, Wood WG, Muller WE, Eckert GP, Müller WE, Eckert GP (2010). Isoprenoids, small GTPases and Alzheimer’s disease. Biochimica et Biophysica Acta.

[CR26] Hottman D, Cheng S, Gram A, LeBlanc K, Yuan LL, Li L (2018). Systemic or forebrain neuron-specific deficiency of geranylgeranyltransferase-1 impairs synaptic plasticity and reduces dendritic spine density. Neuroscience.

[CR27] Hu H-T, Shih P-Y, Shih Y-T, Hsueh Y-P (2016). The involvement of neuron-specific factors in dendritic spinogenesis: Molecular regulation and association with neurological disorders. Neural Plasticity.

[CR28] Jang S-W, Liu X, Yepes M, Shepherd KR, Miller GW, Liu Y (2010). A selective TrkB agonist with potent neurotrophic activities by 7,8-dihydroxyflavone. Proceedings of the National Academy of Sciences of the United States of America.

[CR29] Kawauchi T, Chihama K, Nabeshima Y, Hoshino M (2003). The in vivo roles of STEF/Tiam1, Rac1 and JNK in cortical neuronal migration. The EMBO Journal.

[CR30] Kiraly DD, Eipper-Mains JE, Mains RE, Eipper BA (2010). Synaptic plasticity, a symphony in GEF. ACS Chemical Neuroscience.

[CR31] Kunda P, Paglini G, Quiroga S, Kosik K, Caceres A (2001). Evidence for the involvement of Tiam1 in axon formation. Journal of Neuroscience.

[CR32] Leeuwen FN, Kain HE, Kammen RA, Michiels F, Kranenburg OW, Collard JG (1997). The guanine nucleotide exchange factor Tiam1 affects neuronal morphology; opposing roles for the small GTPases Rac and Rho. Journal of Cell Biology.

[CR33] Lewin GR, Barde YA (1996). Physiology of the neurotrophins. Annual Review of Neuroscience.

[CR34] Li L, Cao D, Kim H, Lester R, Fukuchi K-I (2006). Simvastatin enhances learning and memory independent of amyloid load in mice. Annals of Neurology.

[CR35] Li X-X, Yang T, Wang N, Zhang L-L, Liu X, Xu Y-M (2020). 7,8-Dihydroxyflavone attenuates alcohol-related behavior in rat models of alcohol consumption via TrkB in the ventral tegmental area. Frontiers in Neuroscience.

[CR36] Li, Z., Sun, C., Zhang, T., Mo, J., Shi, Q., Zhang, X., et al. (2013). Geranylgeranyltransferase I mediates BDNF-induced synaptogenesis. *Journal of Neurochemistry*. Retrieved from http://www.ncbi.nlm.nih.gov/entrez/query.fcgi?db=pubmed&cmd=Retrieve&dopt=AbstractPlus&list_uids=23534605. 10.1111/jnc.12249 23534605

[CR37] Liu C, Chan CB, Ye K (2016). 7,8-dihydroxyflavone, a small molecular TrkB agonist, is useful for treating various BDNF-implicated human disorders. Translational Neurodegeneration.

[CR38] Mans RA, Chowdhury N, Cao D, McMahon LL, Li L (2010). Simvastatin enhances hippocampal long-term potentiation in C57BL/6 mice. Neuroscience.

[CR39] Mans, R. A., McMahon, L. L., & Li, L. (2012). Simvastatin-mediated enhancement of long-term potentiation is driven by farnesyl-pyrophosphate depletion and inhibition of farnesylation. *Neuroscience*, *202*, 1–9. Retrieved from http://linkinghub.elsevier.com/retrieve/pii/S030645221101387X. 10.1016/j.neuroscience.2011.12.007 PMC3626073 22192838

[CR40] McTaggart SJ (2005). Isoprenylated proteins. Cellular and Molecular Life Sciences: CMLS.

[CR41] Miquel, K., Pradines, A., Tercé, F., Selmi, S., Favre, G., Terce, F., et al. (1998). Competitive inhibition of choline phosphotransferase by geranylgeraniol and farnesol inhibits phosphatidylcholine synthesis and induces apoptosis in human lung adenocarcinoma A549 cells. *The Journal of Biological Chemistry*, *273*(40), 26179–26186. Retrieved from http://www.ncbi.nlm.nih.gov/pubmed/9748300. 10.1074/jbc.273.40.26179 9748300

[CR42] Miyamoto Y, Yamauchi J, Tanoue A, Wu C, Mobley WC (2006). TrkB binds and tyrosine-phosphorylates Tiam1, leading to activation of Rac1 and induction of changes in cellular morphology. Proceedings of the National Academy of Sciences of the United States of America.

[CR43] Morrison, J. H., & Baxter, M. G. (2012). The ageing cortical synapse: Hallmarks and implications for cognitive decline. *Nature Reviews Neuroscience*, *13*(4), 240–250. Retrieved from http://www.ncbi.nlm.nih.gov/entrez/query.fcgi?db=pubmed&cmd=Retrieve&dopt=AbstractPlus&list_uids=22395804. 10.1038/nrn3200 PMC3592200 22395804

[CR44] Mostany R, Anstey JE, Crump KL, Maco B, Knott G, Portera-Cailliau C (2013). Altered synaptic dynamics during normal brain aging. The Journal of Neuroscience: The Official Journal of the Society for Neuroscience.

[CR45] Newey SE, Velamoor V, Govek EE, Van Aelst L (2005). Rho GTPases, dendritic structure, and mental retardation. Journal of Neurobiology.

[CR46] Nie S, Ma K, Sun M, Lee M, Tan Y, Chen G (2019). 7,8-dihydroxyflavone protects nigrostriatal dopaminergic neurons from rotenone-induced neurotoxicity in rodents. Parkinson’s Disease.

[CR47] Park H, Poo M (2013). Neurotrophin regulation of neural circuit development and function. Nature Reviews Neuroscience.

[CR48] Petralia RS, Mattson MP, Yao PJ (2014). Communication breakdown: The impact of ageing on synapse structure. Ageing Research Reviews.

[CR49] Rosenthal A, Goeddel DV, Nguyen T, Martin E, Burton LE, Shih A (1991). Primary structure and biological activity of human brain-derived neurotrophic factor. Endocrinology.

[CR50] Saetre P, Jazin E, Emilsson L (2011). Age-related changes in gene expression are accelerated in Alzheimer&apos;s disease. Synapse (New York, N. Y.).

[CR51] Saha RN, Liu X, Pahan K (2006). Up-regulation of BDNF in astrocytes by TNF-alpha: a case for the neuroprotective role of cytokine. Journal of Neuroimmune Pharmacology: The Official Journal of the Society on NeuroImmune Pharmacology.

[CR52] Samuel, F., & Hynds, D. L. (2010). RHO GTPase Signaling for Axon Extension: Is Prenylation Important? *Molecular Neurobiology*, *42*(2), 133–142. Retrieved from http://www.ncbi.nlm.nih.gov/entrez/query.fcgi?db=pubmed&cmd=Retrieve&dopt=AbstractPlus&list_uids=20878268. 10.1007/s12035-010-8144-2 20878268

[CR53] Schmidt A, Hall A (2002). Guanine nucleotide exchange factors for Rho GTPases: Turning on the switch. Genes & Development.

[CR54] Schuman, E. M. (1999). Neurotrophin regulation of synaptic transmission. *Current Opinion in Neurobiology*, *9*(1), 105–109. isi:000078748600012. 10.1016/s0959-4388(99)80013-0 10072368

[CR55] Shetty MS, Sajikumar S (2017). ‘Tagging’ along memories in aging: Synaptic tagging and capture mechanisms in the aged hippocampus. Ageing Research Reviews.

[CR56] Skaper SD (2005). Neuronal growth-promoting and inhibitory cues in neuroprotection and neuroregeneration. Annals of the New York Academy of Sciences.

[CR57] Stettler O, Moya KL, Zahraoui A, Tavitian B (1994). Developmental changes in the localization of the synaptic vesicle protein rab3A in rat brain. Neuroscience.

[CR58] Sun T, Chen S, Huang H, Li T, Yang W, Liu L (2017). Metabolic profile study of 7, 8-dihydroxyflavone in monkey plasma using high performance liquid chromatography-tandem mass spectrometry. Journal of Chromatography B, Analytical Technologies in the Biomedical and Life Sciences.

[CR59] Tolias KF, Duman JG, Um K (2011). Control of synapse development and plasticity by Rho GTPase regulatory proteins. Progress in Neurobiology.

[CR60] Tsai T, Klausmeyer A, Conrad R, Gottschling C, Leo M, Faissner A, Wiese S (2013). 7,8-Dihydroxyflavone leads to survival of cultured embryonic motoneurons by activating intracellular signaling pathways. Molecular and Cellular Neurosciences.

[CR100] Wong J, Rothmond DA, Webster MJ, Weickert CS (2013). Increases in two truncated TrkB isoforms in the prefrontal cortex of people with schizophrenia. Schizophrenia Bulletin.

[CR61] Ye X, Carew TJ (2010). Small G protein signaling in neuronal plasticity and memory formation: the specific role of ras family proteins. Neuron.

[CR62] Zeng Y, Lv F, Li L, Yu H, Dong M, Fu Q (2012). 7,8-dihydroxyflavone rescues spatial memory and synaptic plasticity in cognitively impaired aged rats. Journal of Neurochemistry.

[CR63] Zhang Z, Liu X, Schroeder JP, Chan C-B, Song M, Yu SP (2014). 7,8-dihydroxyflavone prevents synaptic loss and memory deficits in a mouse model of Alzheimer’s disease. Neuropsychopharmacology: Official Publication of the American College of Neuropsychopharmacology.

[CR64] Zhou P, Porcionatto M, Pilapil M, Chen Y, Choi Y, Tolias KF (2007). Polarized signaling endosomes coordinate BDNF-induced chemotaxis of cerebellar precursors. Neuron.

[CR65] Zhou X-PP, Wu K-YY, Liang B, Fu X-QQ, Luo Z-GG (2008). TrkB-mediated activation of geranylgeranyltransferase I promotes dendritic morphogenesis. Proceedings of the National Academy of Sciences of the United States of America.

[CR66] Zhou, Y., Suram, A., Venugopal, C., Prakasam, A., Lin, S., Su, Y., et al. (2008). Geranylgeranyl pyrophosphate stimulates gamma-secretase to increase the generation of Abeta and APP-CTFgamma. *The FASEB Journal*, *22*(1), 47–54. Retrieved from http://www.ncbi.nlm.nih.gov/entrez/query.fcgi?db=pubmed&cmd=Retrieve&dopt=AbstractPlus&list_uids=17666454. 10.1096/fj.07-8175com PMC2859886 17666454

